# Promising Effects of Duck Vaccination against Highly Pathogenic Avian Influenza, France, 2023–2024

**DOI:** 10.3201/eid3107.241445

**Published:** 2025-07

**Authors:** Claire Guinat, Lisa Fourtune, Sébastien Lambert, Eva Martin, Guillaume Gerbier, Andrea Jimenez Pellicer, Jean-Luc Guérin, Timothée Vergne

**Affiliations:** Interactions Hôtes-Agents Pathogènes, Université de Toulouse, INRAE, ENVT, Toulouse, France (C. Guinat, L. Fourtune, S. Lambert, E. Martin, J.-L. Guérin, T. Vergne); Direction Générale de l’Alimentation, Ministère de l’Agriculture et de la Souveraineté Alimentaire, Paris, France (G. Gerbier, A. Jimenez Pellicer)

**Keywords:** HPAI, influenza, zoonoses, respiratory infections, viruses, epidemiology, vaccination, modeling, surveillance, France

## Abstract

Highly pathogenic avian influenza causes substantial poultry losses and zoonotic concerns globally. Duck vaccination against highly pathogenic avian influenza began in France in October 2023. Our assessment predicted that 314–756 outbreaks were averted in 2023–2024, representing a 96%–99% reduction in epizootic size, likely attributable to vaccination.

Highly pathogenic avian influenza (HPAI) H5 viruses of clade 2.3.4.4b continue to affect diverse regions and species worldwide. Since 2020, this ongoing panzootic has reached unprecedented scale, causing the death or culling of >130 million poultry across 67 countries, substantially threatening food security (*1*). Mass mortality in wild birds and spillover to >48 mammal species across 26 countries have raised conservation and zoonotic concerns (*2*).

Although most countries rely on poultry depopulation and movement restrictions to control HPAI, France recently implemented preventive vaccination (*3*). Since October 2023, domestic ducks in the production stage are vaccinated with the Volvac B.E.S.T. AI+ND vaccine (Boehringer Ingelheim, https://www.boehringer-ingelheim.com), administered at 10 and 28 days, and, in high-risk zones and during winter, a third dose at 56 days (*4*). In May 2024, the campaign expanded to include the RESPONS AI H5 vaccine (Ceva Animal Health, https://www.ceva.us). Vaccinating breeder ducks remains optional. As of July 1, 2024, >35 million ducks had received 2 doses and 1.5 million had received 3 doses (*4*).

In 2023–2024, only 10 HPAI H5 poultry outbreaks were reported, substantially reduced from 1,374 in 2021–2022 and 396 in 2022–2023 (Figure, panels A, B). Those 10 outbreaks (6 in turkeys, 3 in ducks, and 1 in chickens) were attributed to >4 primary introductions. The infected duck farms shared similar viruses, supporting lateral transmission: 2 occurred in vaccinated flocks with suboptimal immune protection or early virus exposure, and 1 occurred in an unvaccinated breeder flock (*5*). In contrast, outbreaks continued in nonvaccinating countries in Europe. Despite encouraging results, the extent to which vaccination contributed to this reduction remains unclear.

**Figure:**
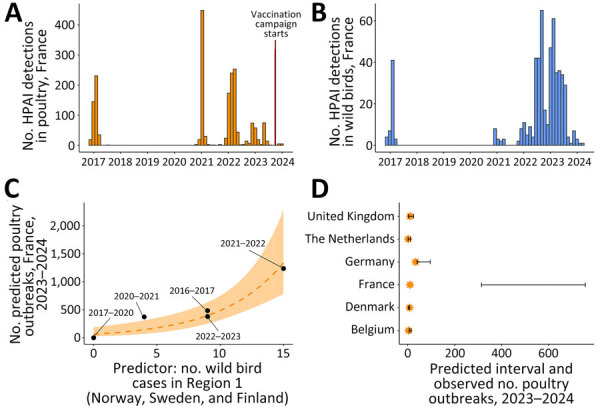
Distribution of and predicted numbers of HPAI H5 cases in study of promising effects of duck vaccination against HPAI, France, 2023–2024. A) Temporal distribution of HPAI H5 clade 2.3.4.4b poultry outbreaks in France. Red vertical line indicates start of duck vaccination campaign (October 1, 2023). B) Temporal distribution of HPAI H5 clade 2.3.4.4b wild bird cases in France. C) Predicted number of HPAI H5 poultry farm outbreaks in France as a function of the predictor variable: number of HPAI H5 wild bird cases in region 1 (Norway, Sweden, Finland). Black dots represent observed number of outbreaks in France in 2016–2023. D) Predicted and observed number of HPAI H5 poultry farm outbreaks in France and in heavily affected, nonvaccinating countries in Europe in 2023–2024. Orange stars represent observed numbers; error bars represent 95% prediction intervals. HPAI, highly pathogenic avian influenza.

We compared the reduction in outbreaks to what would have been expected based on historical outbreak patterns and external infection pressure and considered potential explanations. We extracted HPAI H5 clade 2.3.4.4b detection data in Europe (2016–2024) from the EMPRES Global Animal Disease Information System (https://empres-i.apps.fao.org). For each epidemiologic year (September 1–August 31), we retrieved poultry outbreak numbers in France. We defined candidate predictors using poultry outbreak and wild bird case numbers from neighboring countries as proxies for external infection pressure, supported by phylogenetic links between circulating viruses in those regions (*6*,*7*). Predictors combined time windows (1–3 months before the first poultry outbreak in France) and regions (region 1 [Norway, Sweden, Finland]; region 2 [Germany, Denmark, The Netherlands, Belgium]; region 3 [United Kingdom, Ireland]; region 4 [Bulgaria, Romania, Hungary, Poland, Czech Republic]) (Appendix Table 1, Figure 1). We assumed consistent surveillance across years, likely valid for poultry because of standardized programs in Europe but less certain for wild birds given the opportunistic nature of passive surveillance. Using quasi-Poisson univariate regressions, we identified the predictor most statistically associated with yearly outbreak numbers in France during the prevaccination period 2016–2023 (p<0.05, pseudo-R^2^>0.80). We then used the 2023–2024 value of that predictor to predict the expected number of outbreaks in France, assuming no changes in mitigation strategies. To validate the method, we applied the same approach to heavily affected and nonvaccinating countries in Europe (Appendix Table 2, Figure 2).

The best predictor of the number of poultry outbreaks in France was the number of wild bird cases in region 1 a month before the first outbreak. That association does not imply direct causation but likely reflects infection pressure and spillover risk. Using this variable, the model predicted 487 (95% CI 314–756) outbreaks in France in 2023–2024 (Figure, panel C), greatly exceeding the 10 observed (96%–99% reduction). By contrast, predictions for other countries closely matched observed numbers, supporting model validity (Figure, panel D). Outbreak numbers in Germany were near the lower prediction bound, possibly reflecting improved biosecurity or changes in poultry population dynamics, which remain to be investigated.

Our findings suggest the reduction in France’s outbreak numbers in 2023–2024 likely resulted from vaccination, an intervention absent in other countries in Europe. Although general declines in wild bird cases might have reduced environmental contamination (*1*), that alone cannot explain the discrepancy in France, because such a trend would be expected elsewhere in Europe (Appendix Figure 1). Moreover, the number of primary virus introductions in France in 2023–2024 (n = 4) remained within the same range as in previous waves (*8*,*9*). Although the duck population declined in 2020–2022 because of previous outbreaks, it increased in 2023 (*5*), ruling out reduced duck population as an explanation. Assuming farm biosecurity and other measures (e.g., movement restrictions and indoor confinement) remained unchanged, vaccination appears the most likely driver of the reduction. Whether duck vaccination might have indirectly protected unvaccinated poultry or other factors (e.g., changes in virus virulence) contributed remain to be investigated (*5*). Given differences in poultry sectors between countries, the vaccination strategy used in France, if applied elsewhere, might not yield similar outcomes. Further modeling of vaccination coverage is needed to better quantify its direct effects. However, the potential of vaccination to reduce HPAI incidence and protect both animal and public health warrants consideration.

Appendix Additional information about the promising effects of duck vaccination against highly pathogenic avian influenza, France, 2023–2024
