# Supplementary material for: Promising Effects of Duck Vaccination against Highly Pathogenic Avian Influenza, France, 2023–2024
Source: Emerg Infect Dis. 2025 Jul;31(7):1468–71. doi: 10.3201/eid3107.241445 (PMC12205464; doi:10.3201/eid3107.241445)
Supplement: Appendix — Additional information about the promising effects of duck vaccination against highly pathogenic avian influenza, France, 2023–2024 [file 24-1445-Techapp-s1.pdf]

Article DOI: https://doi.org/10.3201/eid3107.241445

EID cannot ensure accessibility for supplementary materials supplied by authors. Readers who have difficulty accessing supplementary content should contact the authors for assistance.

Promising Effects of Duck Vaccination against Highly Pathogenic Avian Influenza, France 2023–2024

Appendix

Appendix Table 1. List of candidate predictors with associated numbers of poultry outbreaks and wild bird cases.

Country	2016–17	2017–18	2018–19	2019–20	2020–21	2021–22	2022–23
**FRANCE**							
**Poultry outbreaks - FR**	**484**	**0**	**0**	**0**	**380**	**1237**	**373**
Domestic - BG, RO, HU, PO, CZ - 1 mo	58	1	5	0	5	29	2
Wild - BG, RO, HU, PO, CZ - 1 mo	10	0	0	0	0	10	0
Domestic - GE, DK, NL, BE - 1 mo	23	0	0	0	18	21	26
Wild - GE, DK, NL, BE - 1 mo	132	0	0	0	311	183	24
Domestic - NW, SW, FN - 1 mo	1	0	0	0	2	1	0
Wild - NW, SW, FN - 1 mo	9	0	0	0	9	15	4
Domestic - UK, IR - 1 mo	0	0	0	0	6	9	17
Wild - UK, IR - 1 mo	0	0	0	0	91	202	1
Domestic - BG, RO, HU, PO, CZ - 2 mo	58	3	13	0	5	29	2
Wild - BG, RO, HU, PO, CZ - 2 mo	11	0	0	0	0	10	0
Domestic - GE, DK, NL, BE - 2 mo	23	0	0	0	20	21	45
Wild - GE, DK, NL, BE - 2 mo	132	1	0	0	417	197	63
Domestic - NW, SW, FN - 2 mo	1	0	0	0	2	1	0
Wild - NW, SW, FN - 2 mo	9	0	0	0	11	23	5
Domestic - UK, IR - 2 mo	0	0	0	0	7	10	27
Wild - UK, IR - 2 mo	0	0	0	0	104	207	56
Domestic - BG, RO, HU, PO, CZ - 3 mo	58	3	14	0	5	29	6
Wild - BG, RO, HU, PO, CZ - 3 mo	11	0	0	0	0	10	1
Domestic - GE, DK, NL, BE - 3 mo	23	0	0	0	20	21	58
Wild - GE, DK, NL, BE - 3 mo	132	1	0	0	417	197	99
Domestic - NW, SW, FN - 3 mo	1	0	0	0	2	1	0
Wild - NW, SW, FN - 3 mo	9	0	0	0	11	29	22
Domestic - UK, IR - 3 mo	0	0	0	0	7	10	32
Wild - UK, IR - 3 mo	0	0	0	0	104	207	73
Domestic - BG, RO, HU, PO, CZ - Nov	76	1	5	0	1	52	36
Wild - BG, RO, HU, PO, CZ - Nov	8	0	0	0	0	14	8
Domestic - GE, DK, NL, BE - Nov	26	0	0	0	19	26	11
Wild - GE, DK, NL, BE - Nov	145	0	0	0	376	214	34
Domestic - NW, SW, FN - Nov	1	0	0	0	2	1	0
Wild - NW, SW, FN - Nov	10	0	0	0	9	13	2
Domestic - UK, IR - Nov	0	0	0	0	3	17	16
Wild - UK, IR - Nov	0	0	0	0	80	229	14
Domestic - BG, RO, HU, PO, CZ - OctNov	76	3	13	0	1	52	36
Wild - BG, RO, HU, PO, CZ - OctNov	12	0	0	0	0	14	8
Domestic - GE, DK, NL, BE - OctNov	26	0	0	0	19	29	53
Wild - GE, DK, NL, BE - OctNov	145	1	0	0	408	268	62
Domestic - NW, SW, FN - OctNov	1	0	0	0	2	1	1
Wild - NW, SW, FN - OctNov	10	0	0	0	10	18	5
Domestic - UK, IR - OctNov	0	0	0	0	4	20	72
Wild - UK, IR - OctNov	0	0	0	0	81	253	34
Domestic - BG, RO, HU, PO, CZ - SepOctNov	76	3	14	0	1	52	38

Country	2016–17	2017–18	2018–19	2019–20	2020–21	2021–22	2022–23
Wild - BG, RO, HU, PO, CZ - SepOctNov	12	0	0	0	0	14	8
Domestic - GE, DK, NL, BE - SepOctNov	26	0	0	0	19	29	78
Wild - GE, DK, NL, BE - SepOctNov	145	1	0	0	408	268	86
Domestic - NW, SW, FN - SepOctNov	1	0	0	0	2	1	1
Wild - NW, SW, FN - SepOctNov	10	0	0	0	10	26	8
Domestic - UK, IR - SepOctNov	0	0	0	0	4	20	86
Wild - UK, IR - SepOctNov	0	0	0	0	81	253	67
**BELGIUM**							
**Poultry outbreaks - BE**	**17**	**0**	**0**	**0**	**2**	**7**	**25**
Domestic - BG, RO, HU, PO, CZ - 1 mo	107	0	1	0	0	54	0
Wild - BG, RO, HU, PO, CZ - 1 mo	109	0	0	0	0	12	0
Domestic - GE, DK, NL, BE - 1 mo	24	0	0	0	16	25	22
Wild - GE, DK, NL, BE - 1 mo	36	0	0	0	354	230	39
Domestic - NW, SW, FN - 1 mo	1	0	0	0	1	1	0
Wild - NW, SW, FN - 1 mo	10	0	0	0	6	7	1
Domestic - UK, IR - 1 mo	6	0	0	0	3	22	12
Wild - UK, IR - 1 mo	21	0	0	0	59	232	55
Domestic - BG, RO, HU, PO, CZ - 2 mo	293	1	10	0	0	54	4
Wild - BG, RO, HU, PO, CZ - 2 mo	113	0	0	0	0	14	0
Domestic - GE, DK, NL, BE - 2 mo	40	0	0	0	16	31	36
Wild - GE, DK, NL, BE - 2 mo	109	0	0	0	356	296	72
Domestic - NW, SW, FN - 2 mo	2	0	0	0	1	1	0
Wild - NW, SW, FN - 2 mo	24	0	0	0	6	18	13
Domestic - UK, IR - 2 mo	7	0	0	0	3	25	17
Wild - UK, IR - 2 mo	26	0	0	0	59	263	71
Domestic - BG, RO, HU, PO, CZ - 3 mo	351	3	13	0	0	54	5
Wild - BG, RO, HU, PO, CZ - 3 mo	123	0	0	0	0	14	1
Domestic - GE, DK, NL, BE - 3 mo	63	0	0	0	16	31	38
Wild - GE, DK, NL, BE - 3 mo	241	1	0	0	356	296	181
Domestic - NW, SW, FN - 3 mo	3	0	0	0	1	1	0
Wild - NW, SW, FN - 3 mo	33	0	0	0	6	26	32
Domestic - UK, IR - 3 mo	7	0	0	0	3	25	18
Wild - UK, IR - 3 mo	26	0	0	0	59	263	95
Domestic - BG, RO, HU, PO, CZ - Nov	76	1	5	0	1	52	36
Wild - BG, RO, HU, PO, CZ - Nov	8	0	0	0	0	14	8
Domestic - GE, DK, NL, BE - Nov	26	0	0	0	19	26	11
Wild - GE, DK, NL, BE - Nov	145	0	0	0	376	214	34
Domestic - NW, SW, FN - Nov	1	0	0	0	2	1	0
Wild - NW, SW, FN - Nov	10	0	0	0	9	13	2
Domestic - UK, IR - Nov	0	0	0	0	3	17	16
Wild - UK, IR - Nov	0	0	0	0	80	229	14
Domestic - BG, RO, HU, PO, CZ - OctNov	76	3	13	0	1	52	36
Wild - BG, RO, HU, PO, CZ - OctNov	12	0	0	0	0	14	8
Domestic - GE, DK, NL, BE - OctNov	26	0	0	0	19	29	53
Wild - GE, DK, NL, BE - OctNov	145	1	0	0	408	268	62
Domestic - NW, SW, FN - OctNov	1	0	0	0	2	1	1
Wild - NW, SW, FN - OctNov	10	0	0	0	10	18	5
Domestic - UK, IR - OctNov	0	0	0	0	4	20	72
Wild - UK, IR - OctNov	0	0	0	0	81	253	34
Domestic - BG, RO, HU, PO, CZ - SepOctNov	76	3	14	0	1	52	38
Wild - BG, RO, HU, PO, CZ - SepOctNov	12	0	0	0	0	14	8
Domestic - GE, DK, NL, BE - SepOctNov	26	0	0	0	19	29	78
Wild - GE, DK, NL, BE - SepOctNov	145	1	0	0	408	268	86
Domestic - NW, SW, FN - SepOctNov	1	0	0	0	2	1	1
Wild - NW, SW, FN - SepOctNov	10	0	0	0	10	26	8
Domestic - UK, IR - SepOctNov	0	0	0	0	4	20	86
Wild - UK, IR - SepOctNov	0	0	0	0	81	253	67
**DENMARK**							
**Poultry outbreaks - DK**	**5**	**0**	**0**	**0**	**17**	**9**	**8**
Domestic - BG, RO, HU, PO, CZ - 1 mo	20	2	9	0	0	0	2
Wild - BG, RO, HU, PO, CZ - 1 mo	6	0	0	0	0	2	0
Domestic - GE, DK, NL, BE - 1 mo	18	0	0	0	11	6	31
Wild - GE, DK, NL, BE - 1 mo	93	1	0	0	335	66	23
Domestic - NW, SW, FN - 1 mo	0	0	0	0	1	0	0
Wild - NW, SW, FN - 1 mo	2	0	0	0	6	11	4
Domestic - UK, IR - 1 mo	0	0	0	0	2	3	26
Wild - UK, IR - 1 mo	0	0	0	0	38	31	1
Domestic - BG, RO, HU, PO, CZ - 2 mo	20	2	12	0	0	0	2

Country	2016–17	2017–18	2018–19	2019–20	2020–21	2021–22	2022–23
Wild - BG, RO, HU, PO, CZ - 2 mo	6	0	0	0	0	2	0
Domestic - GE, DK, NL, BE - 2 mo	18	0	0	0	11	6	52
Wild - GE, DK, NL, BE - 2 mo	93	1	0	0	335	66	61
Domestic - NW, SW, FN - 2 mo	0	0	0	0	1	0	0
Wild - NW, SW, FN - 2 mo	2	0	0	0	6	19	4
Domestic - UK, IR - 2 mo	0	0	0	0	2	3	37
Wild - UK, IR - 2 mo	0	0	0	0	38	31	54
Domestic - BG, RO, HU, PO, CZ - 3 mo	20	2	12	0	0	0	6
Wild - BG, RO, HU, PO, CZ - 3 mo	6	0	0	0	0	2	0
Domestic - GE, DK, NL, BE - 3 mo	18	0	0	0	11	6	67
Wild - GE, DK, NL, BE - 3 mo	93	2	0	0	335	66	95
Domestic - NW, SW, FN - 3 mo	0	0	0	0	1	0	0
Wild - NW, SW, FN - 3 mo	2	0	0	0	6	29	17
Domestic - UK, IR - 3 mo	0	0	0	0	2	3	43
Wild - UK, IR - 3 mo	0	0	0	0	38	31	72
Domestic - BG, RO, HU, PO, CZ - Nov	76	1	5	0	1	52	36
Wild - BG, RO, HU, PO, CZ - Nov	8	0	0	0	0	14	8
Domestic - GE, DK, NL, BE - Nov	26	0	0	0	19	26	11
Wild - GE, DK, NL, BE - Nov	145	0	0	0	376	214	34
Domestic - NW, SW, FN - Nov	1	0	0	0	2	1	0
Wild - NW, SW, FN - Nov	10	0	0	0	9	13	2
Domestic - UK, IR - Nov	0	0	0	0	3	17	16
Wild - UK, IR - Nov	0	0	0	0	80	229	14
Domestic - BG, RO, HU, PO, CZ - OctNov	76	3	13	0	1	52	36
Wild - BG, RO, HU, PO, CZ - OctNov	12	0	0	0	0	14	8
Domestic - GE, DK, NL, BE - OctNov	26	0	0	0	19	29	53
Wild - GE, DK, NL, BE - OctNov	145	1	0	0	408	268	62
Domestic - NW, SW, FN - OctNov	1	0	0	0	2	1	1
Wild - NW, SW, FN - OctNov	10	0	0	0	10	18	5
Domestic - UK, IR - OctNov	0	0	0	0	4	20	72
Wild - UK, IR - OctNov	0	0	0	0	81	253	34
Domestic - BG, RO, HU, PO, CZ - SepOctNov	76	3	14	0	1	52	38
Wild - BG, RO, HU, PO, CZ - SepOctNov	12	0	0	0	0	14	8
Domestic - GE, DK, NL, BE - SepOctNov	26	0	0	0	19	29	78
Wild - GE, DK, NL, BE - SepOctNov	145	1	0	0	408	268	86
Domestic - NW, SW, FN - SepOctNov	1	0	0	0	2	1	1
Wild - NW, SW, FN - SepOctNov	10	0	0	0	10	26	8
Domestic - UK, IR - SepOctNov	0	0	0	0	4	20	86
Wild - UK, IR - SepOctNov	0	0	0	0	81	253	67
**GERMANY**							
**Poultry outbreaks - GE**	**99**	**0**	**0**	**3**	**222**	**84**	**85**
Domestic - BG, RO, HU, PO, CZ - 1 mo	2	1	2	17	0	0	0
Wild - BG, RO, HU, PO, CZ - 1 mo	5	0	0	1	0	0	0
Domestic - GE, DK, NL, BE - 1 mo	3	0	0	1	2	2	14
Wild - GE, DK, NL, BE - 1 mo	19	0	0	0	106	21	38
Domestic - NW, SW, FN - 1 mo	0	0	0	0	0	0	0
Wild - NW, SW, FN - 1 mo	0	0	0	0	2	8	7
Domestic - UK, IR - 1 mo	0	0	0	0	1	1	10
Wild - UK, IR - 1 mo	0	0	0	0	13	5	29
Domestic - BG, RO, HU, PO, CZ - 2 mo	2	3	11	27	0	0	4
Wild - BG, RO, HU, PO, CZ - 2 mo	5	0	0	1	0	0	1
Domestic - GE, DK, NL, BE - 2 mo	3	0	0	1	2	2	25
Wild - GE, DK, NL, BE - 2 mo	19	1	0	0	106	21	94
Domestic - NW, SW, FN - 2 mo	0	0	0	0	0	0	0
Wild - NW, SW, FN - 2 mo	0	0	0	0	2	11	22
Domestic - UK, IR - 2 mo	0	0	0	0	1	1	13
Wild - UK, IR - 2 mo	0	0	0	0	13	5	56
Domestic - BG, RO, HU, PO, CZ - 3 mo	2	3	14	27	0	0	8
Wild - BG, RO, HU, PO, CZ - 3 mo	5	0	0	1	0	0	5
Domestic - GE, DK, NL, BE - 3 mo	3	0	0	1	2	2	32
Wild - GE, DK, NL, BE - 3 mo	19	1	0	0	106	22	199
Domestic - NW, SW, FN - 3 mo	0	0	0	0	0	0	0
Wild - NW, SW, FN - 3 mo	0	0	0	0	2	22	50
Domestic - UK, IR - 3 mo	0	0	0	0	1	1	15
Wild - UK, IR - 3 mo	0	0	0	0	13	6	93
Domestic - BG, RO, HU, PO, CZ - Nov	76	1	5	0	1	52	36
Wild - BG, RO, HU, PO, CZ - Nov	8	0	0	0	0	14	8
Domestic - GE, DK, NL, BE - Nov	26	0	0	0	19	26	11
Wild - GE, DK, NL, BE - Nov	145	0	0	0	376	214	34

Country	2016–17	2017–18	2018–19	2019–20	2020–21	2021–22	2022–23
Domestic - NW, SW, FN - Nov	1	0	0	0	2	1	0
Wild - NW, SW, FN - Nov	10	0	0	0	9	13	2
Domestic - UK, IR - Nov	0	0	0	0	3	17	16
Wild - UK, IR - Nov	0	0	0	0	80	229	14
Domestic - BG, RO, HU, PO, CZ - OctNov	76	3	13	0	1	52	36
Wild - BG, RO, HU, PO, CZ - OctNov	12	0	0	0	0	14	8
Domestic - GE, DK, NL, BE - OctNov	26	0	0	0	19	29	53
Wild - GE, DK, NL, BE - OctNov	145	1	0	0	408	268	62
Domestic - NW, SW, FN - OctNov	1	0	0	0	2	1	1
Wild - NW, SW, FN - OctNov	10	0	0	0	10	18	5
Domestic - UK, IR - OctNov	0	0	0	0	4	20	72
Wild - UK, IR - OctNov	0	0	0	0	81	253	34
Domestic - BG, RO, HU, PO, CZ - SepOctNov	76	3	14	0	1	52	38
Wild - BG, RO, HU, PO, CZ - SepOctNov	12	0	0	0	0	14	8
Domestic - GE, DK, NL, BE - SepOctNov	26	0	0	0	19	29	78
Wild - GE, DK, NL, BE - SepOctNov	145	1	0	0	408	268	86
Domestic - NW, SW, FN - SepOctNov	1	0	0	0	2	1	1
Wild - NW, SW, FN - SepOctNov	10	0	0	0	10	26	8
Domestic - UK, IR - SepOctNov	0	0	0	0	4	20	86
Wild - UK, IR - SepOctNov	0	0	0	0	81	253	67
**NETHERLANDS**							
**Poultry outbreaks - NL**	**9**	**0**	**0**	**0**	**9**	**44**	**29**
Domestic - BG, RO, HU, PO, CZ - 1 mo	58	2	9	0	0	0	0
Wild - BG, RO, HU, PO, CZ - 1 mo	10	0	0	0	0	0	0
Domestic - GE, DK, NL, BE - 1 mo	23	0	0	0	2	3	13
Wild - GE, DK, NL, BE - 1 mo	132	1	0	0	106	29	37
Domestic - NW, SW, FN - 1 mo	1	0	0	0	0	0	0
Wild - NW, SW, FN - 1 mo	9	0	0	0	2	4	7
Domestic - UK, IR - 1 mo	0	0	0	0	1	1	9
Wild - UK, IR - 1 mo	0	0	0	0	13	11	30
Domestic - BG, RO, HU, PO, CZ - 2 mo	58	2	12	0	0	0	4
Wild - BG, RO, HU, PO, CZ - 2 mo	11	0	0	0	0	0	1
Domestic - GE, DK, NL, BE - 2 mo	23	0	0	0	2	3	23
Wild - GE, DK, NL, BE - 2 mo	132	1	0	0	106	29	105
Domestic - NW, SW, FN - 2 mo	1	0	0	0	0	0	0
Wild - NW, SW, FN - 2 mo	9	0	0	0	2	12	22
Domestic - UK, IR - 2 mo	0	0	0	0	1	1	12
Wild - UK, IR - 2 mo	0	0	0	0	13	11	57
Domestic - BG, RO, HU, PO, CZ - 3 mo	58	2	12	0	0	0	8
Wild - BG, RO, HU, PO, CZ - 3 mo	11	0	0	0	0	0	5
Domestic - GE, DK, NL, BE - 3 mo	23	0	0	0	2	3	30
Wild - GE, DK, NL, BE - 3 mo	132	2	0	0	106	29	201
Domestic - NW, SW, FN - 3 mo	1	0	0	0	0	0	0
Wild - NW, SW, FN - 3 mo	9	0	0	0	2	23	52
Domestic - UK, IR - 3 mo	0	0	0	0	1	1	13
Wild - UK, IR - 3 mo	0	0	0	0	13	12	93
Domestic - BG, RO, HU, PO, CZ - Nov	76	1	5	0	1	52	36
Wild - BG, RO, HU, PO, CZ - Nov	8	0	0	0	0	14	8
Domestic - GE, DK, NL, BE - Nov	26	0	0	0	19	26	11
Wild - GE, DK, NL, BE - Nov	145	0	0	0	376	214	34
Domestic - NW, SW, FN - Nov	1	0	0	0	2	1	0
Wild - NW, SW, FN - Nov	10	0	0	0	9	13	2
Domestic - UK, IR - Nov	0	0	0	0	3	17	16
Wild - UK, IR - Nov	0	0	0	0	80	229	14
Domestic - BG, RO, HU, PO, CZ - OctNov	76	3	13	0	1	52	36
Wild - BG, RO, HU, PO, CZ - OctNov	12	0	0	0	0	14	8
Domestic - GE, DK, NL, BE - OctNov	26	0	0	0	19	29	53
Wild - GE, DK, NL, BE - OctNov	145	1	0	0	408	268	62
Domestic - NW, SW, FN - OctNov	1	0	0	0	2	1	1
Wild - NW, SW, FN - OctNov	10	0	0	0	10	18	5
Domestic - UK, IR - OctNov	0	0	0	0	4	20	72
Wild - UK, IR - OctNov	0	0	0	0	81	253	34
Domestic - BG, RO, HU, PO, CZ - SepOctNov	76	3	14	0	1	52	38
Wild - BG, RO, HU, PO, CZ - SepOctNov	12	0	0	0	0	14	8
Domestic - GE, DK, NL, BE - SepOctNov	26	0	0	0	19	29	78
Wild - GE, DK, NL, BE - SepOctNov	145	1	0	0	408	268	86
Domestic - NW, SW, FN - SepOctNov	1	0	0	0	2	1	1
Wild - NW, SW, FN - SepOctNov	10	0	0	0	10	26	8
Domestic - UK, IR - SepOctNov	0	0	0	0	4	20	86

Country	2016–17	2017–18	2018–19	2019–20	2020–21	2021–22	2022–23
Wild - UK, IR - SepOctNov	0	0	0	0	81	253	67
**UNITED KINGDOM**							
**Poultry outbreaks - UK**	**13**	**0**	**0**	**0**	**18**	**79**	**123**
Domestic - BG, RO, HU, PO, CZ - 1 mo	195	2	10	0	0	0	0
Wild - BG, RO, HU, PO, CZ - 1 mo	7	0	0	0	0	0	0
Domestic - GE, DK, NL, BE - 1 mo	22	0	0	0	0	0	14
Wild - GE, DK, NL, BE - 1 mo	137	1	0	0	3	8	38
Domestic - NW, SW, FN - 1 mo	1	0	0	0	0	0	0
Wild - NW, SW, FN - 1 mo	17	0	0	0	0	8	7
Domestic - UK, IR - 1 mo	1	0	0	0	1	1	10
Wild - UK, IR - 1 mo	0	0	0	0	0	4	29
Domestic - BG, RO, HU, PO, CZ - 2 mo	200	2	13	0	0	0	4
Wild - BG, RO, HU, PO, CZ - 2 mo	13	0	0	0	0	0	1
Domestic - GE, DK, NL, BE - 2 mo	28	0	0	0	0	0	25
Wild - GE, DK, NL, BE - 2 mo	185	1	0	0	3	8	94
Domestic - NW, SW, FN - 2 mo	1	0	0	0	0	0	0
Wild - NW, SW, FN - 2 mo	18	0	0	0	0	15	22
Domestic - UK, IR - 2 mo	1	0	0	0	1	1	13
Wild - UK, IR - 2 mo	0	0	0	0	0	4	56
Domestic - BG, RO, HU, PO, CZ - 3 mo	200	2	13	0	0	0	8
Wild - BG, RO, HU, PO, CZ - 3 mo	13	0	0	0	0	0	5
Domestic - GE, DK, NL, BE - 3 mo	28	0	0	0	0	0	32
Wild - GE, DK, NL, BE - 3 mo	185	2	0	0	3	9	199
Domestic - NW, SW, FN - 3 mo	1	0	0	0	0	0	0
Wild - NW, SW, FN - 3 mo	18	0	0	0	0	22	50
Domestic - UK, IR - 3 mo	1	0	0	0	1	1	15
Wild - UK, IR - 3 mo	0	0	0	0	0	6	93
Domestic - BG, RO, HU, PO, CZ - Nov	76	1	5	0	1	52	36
Wild - BG, RO, HU, PO, CZ - Nov	8	0	0	0	0	14	8
Domestic - GE, DK, NL, BE - Nov	26	0	0	0	19	26	11
Wild - GE, DK, NL, BE - Nov	145	0	0	0	376	214	34
Domestic - NW, SW, FN - Nov	1	0	0	0	2	1	0
Wild - NW, SW, FN - Nov	10	0	0	0	9	13	2
Domestic - UK, IR - Nov	0	0	0	0	3	17	16
Wild - UK, IR - Nov	0	0	0	0	80	229	14
Domestic - BG, RO, HU, PO, CZ - OctNov	76	3	13	0	1	52	36
Wild - BG, RO, HU, PO, CZ - OctNov	12	0	0	0	0	14	8
Domestic - GE, DK, NL, BE - OctNov	26	0	0	0	19	29	53
Wild - GE, DK, NL, BE - OctNov	145	1	0	0	408	268	62
Domestic - NW, SW, FN - OctNov	1	0	0	0	2	1	1
Wild - NW, SW, FN - OctNov	10	0	0	0	10	18	5
Domestic - UK, IR - OctNov	0	0	0	0	4	20	72
Wild - UK, IR - OctNov	0	0	0	0	81	253	34
Domestic - BG, RO, HU, PO, CZ - SepOctNov	76	3	14	0	1	52	38
Wild - BG, RO, HU, PO, CZ - SepOctNov	12	0	0	0	0	14	8
Domestic - GE, DK, NL, BE - SepOctNov	26	0	0	0	19	29	78
Wild - GE, DK, NL, BE - SepOctNov	145	1	0	0	408	268	86
Domestic - NW, SW, FN - SepOctNov	1	0	0	0	2	1	1
Wild - NW, SW, FN - SepOctNov	10	0	0	0	10	26	8
Domestic - UK, IR - SepOctNov	0	0	0	0	4	20	86
Wild - UK, IR - SepOctNov	0	0	0	0	81	253	67

Predictor names have been abbreviated as follows: Domestic = poultry outbreaks, Wild = wild bird cases. Country abbreviations: BG = Bulgaria, RO = Romania, HU = Hungary, PO = Poland, CZ = Czech Republic, GE = Germany, DK = Denmark, NL = Netherlands, BE = Belgium, NW = Norway, SW = Sweden, FN = Finland, UK = United Kingdom, IR = Ireland. Time abbreviations: 1mo/2mo/3mo = 1, 2, or 3 mo before the first reported outbreak in the studied country; Sep = September, Oct = October, Nov = November.

Appendix Table 2. Best model results for each studied country.

Model	Model p-value	Pseudo r^2	β_0	p-value	β_1	p-value
Poultry outbreaks - FR ~ Wild - NW, SW, FN - 1 mo	<0.001	0.81	4.17	<0.001	0.20	<0.001
Poultry outbreaks - BE ~ Wild - NW, SW, FN - 3 mo	<0.001	0.92	−1.19	0.25	0.13	0.007
Poultry outbreaks - DK ~ Domestic - NW, SW, FN - OctNov	<0.001	0.76	0.17	0.75	1.42	0.008
Poultry outbreaks - GE ~ Domestic - NW, SW, FN - OctNov	<0.001	0.82	2.68	0.001	1.44	0.004
Poultry outbreaks - NL ~ Domestic - UK, IR - Nov	<0.001	0.79	1.11	0.11	0.15	0.01
Poultry outbreaks - UK ~ Domestic - UK, IR - Nov	<0.001	0.84	1.70	0.04	0.18	0.007

Predictor names have been abbreviated as follows: Domestic = poultry outbreaks, Wild = wild bird cases. Country abbreviations: BG = Bulgaria, RO = Romania, HU = Hungary, PO = Poland, CZ = Czech Republic, GE = Germany, DK = Denmark, NL = Netherlands, BE = Belgium, NW = Norway, SW = Sweden, FN = Finland, UK = United Kingdom, IR = Ireland. Time abbreviations: 1mo/2mo/3mo = 1, 2, or 3 mo before the first reported outbreak in the studied country; Sep = September, Oct = October, Nov = November.

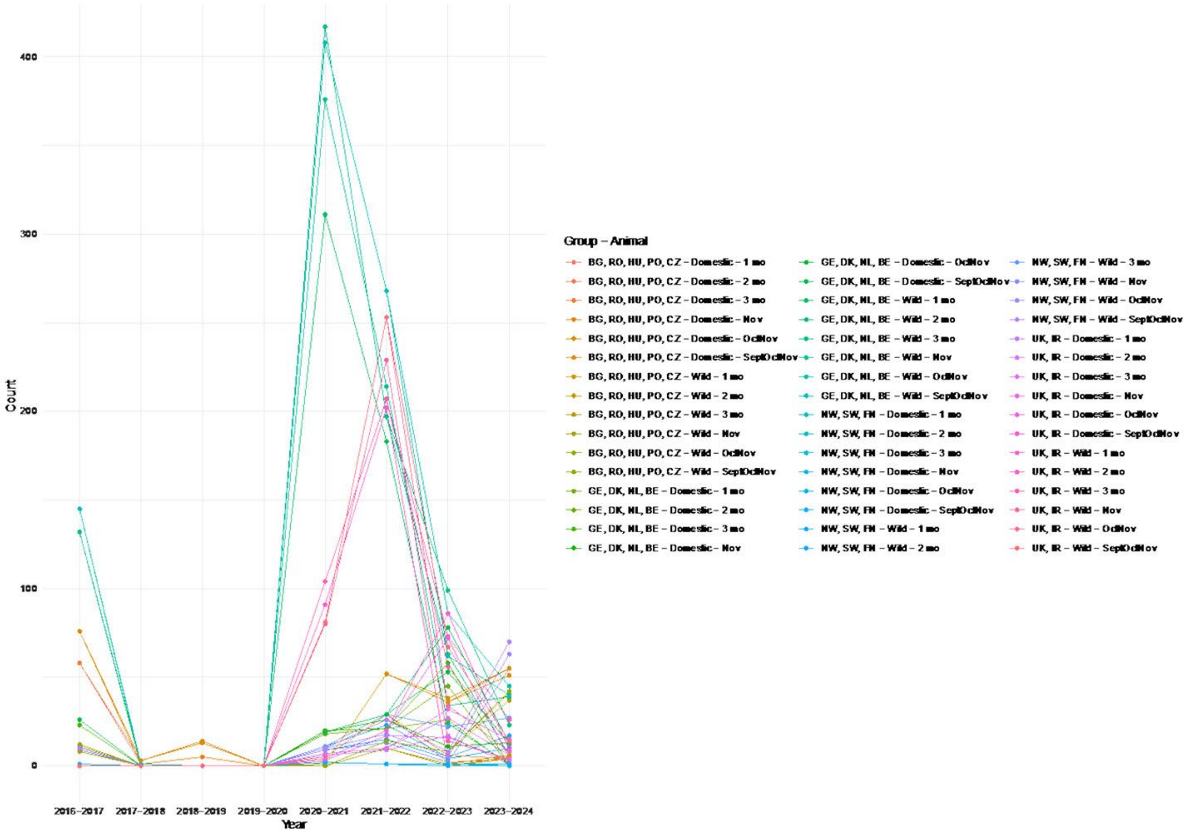

Appendix Figure 1. Temporal distribution of candidate predictors with associated numbers of poultry outbreaks and wild bird cases. Predictor names have been abbreviated as follows: Domestic = poultry outbreaks, Wild = wild bird cases. Country abbreviations: BG = Bulgaria, RO = Romania, HU = Hungary, PO = Poland, CZ = Czech Republic, GE = Germany, DK = Denmark, NL = Netherlands, BE = Belgium, NW = Norway, SW = Sweden, FN = Finland, UK = United Kingdom, IR = Ireland. Time abbreviations: 1mo/2mo/3mo = 1, 2, or 3 months before the first outbreak in the studied country; Sep = September, Oct = October, Nov = November.

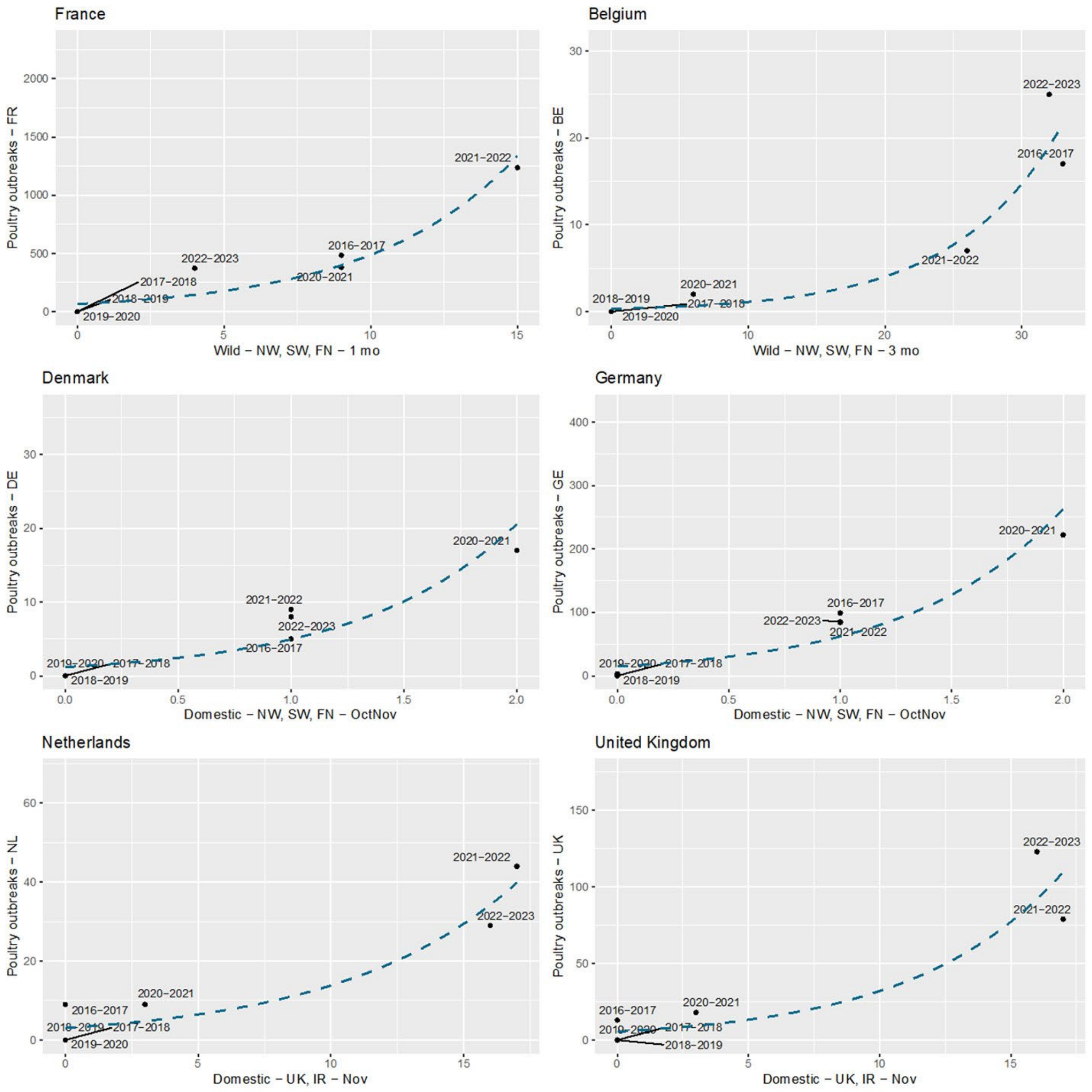

Appendix Figure 2. Predicted number of HPAI H5 poultry farm outbreaks for each studied country as a function of the predictor most statistically associated. Black dots represent the observed number of outbreaks for each epidemiologic year. Predictor names have been abbreviated as follows: Domestic = poultry outbreaks, Wild = wild bird cases. Country abbreviations: BG = Bulgaria, RO = Romania, HU = Hungary, PO = Poland, CZ = Czech Republic, GE = Germany, DK = Denmark, NL = Netherlands, BE = Belgium, NW = Norway, SW = Sweden, FN = Finland, UK = United Kingdom, IR = Ireland. Time abbreviations: 1mo/2mo/3mo = 1, 2, or 3 months before the first outbreak in the studied country; Sep = September, Oct = October, Nov = November.
